# Outcome of a Community-Based Oral Health Promotion Project on Primary Schoolchildren's Oral Hygiene Habits

**DOI:** 10.1155/2013/485741

**Published:** 2013-11-12

**Authors:** Heidi Halonen, Paula Pesonen, Liisa Seppä, Eija Peltonen, Leo Tjäderhane, Vuokko Anttonen

**Affiliations:** ^1^Faculty of Medicine, University of Oulu, 90014 Oulu, Finland; ^2^Middle Finland Hospital District, Finland; ^3^Oulu University Hospital, Finland; ^4^Department of Pedodontics, Cariology and Endodontology, University of Oulu, P.O. Box 5281, 90014 Oulu, Finland

## Abstract

The aim of this study was to evaluate the effect of a school-based intervention project conducted in a mid-sized Finnish city, Laukaa on schoolchildren's oral health behavior. *Material and Methods*. In the intervention, all children received dental education and some of the 7–12-year-old schoolchildren received individual tooth brushing instructions by a dental nurse in 2009-2010. Parents were present at the instruction sessions. In 2009 and 2010, all the children answered a questionnaire or an oral hygienist on their oral health behavior without identification. *Results*. Tooth brushing frequency increased significantly among the schoolchildren between the years 2009 (61.2%) and 2010 (65%) (*P* < 0.05); more so among younger children (7–10-year-olds) compared to the older ones (11-12-year-olds). The 2010 results showed a slight trend of decreasing tooth brushing frequency by age both among girls and boys. Younger children got significantly more often parental help or reminding. The girls brushed their teeth significantly more frequently (71.9%) than boys (57.0%). *Conclusions*. Our findings indicate that oral health intervention can be beneficial on health behavior especially for children at low grades. All children, 11 to 12 years of age, especially boys, need continuous health promotion.

## 1. Introduction

According to a WHO survey conducted in 2005/2006, Finnish schoolchildren's tooth brushing frequency was one of the lowest in Europe [1]. Thirty-seven percent of Finnish 11-year-old boys and 55% of girls reported brushing their teeth more frequently than once a day. The figures were similar among 13- and 15-year-olds (boys 35% and 39% and girls 55% and 61%, resp.). All values were clearly below the European mean values: 11-year-olds 56% and 67%, 13-year-olds 55% and 69%, and 15-year-olds 54% and 74% for boys and girls, respectively. Only children in Lithuania, Greece, Turkey, and Malta brushed their teeth more seldom than the Finns. Children in Switzerland and in Finland's neighboring country Sweden brushed their teeth most often [1]. There are no statistics of tooth brushing frequency of children at lower grades.

The time of eruption of molars, particularly the first permanent molars, is considered as a time of high caries risk for decaying [2–4]. It has been reported that intensive dental care at eruption time has resulted in reduction in the amount of visible plaque, need for sealants and fillings, and consequently, amount of frequent recalls. An example of an excellent outcome of oral health promotion comes from Nexø, Denmark. In Nexø, a community-based program was designed and launched during period 1987-1988 to improve oral health by focusing on nonoperative caries treatment of children and adolescents aged 0–18 years and improving their self-care, that is, tooth brushing, especially at times of tooth eruption [4]. DMFT values in the Nexø community have sunk well below the national average during the past decades, that is, after the intervention started [5, 6]. The total costs of the dental services decreased approximately by 15% during the period from 1987 to 1999 [6]. In Finland, after a community-based oral health intervention on schoolchildren, a survey revealed that tooth brushing frequency, knowledge, and attitudes of schoolchildren in terms of oral health improved. However, it was concluded that to have an optimum outcome, oral health promotion should be a continuous process rather than a short-term intervention [7].

In Finland, dental care is free up to the age of 18 and children are invited to regular examinations by a dentist or a dental assistant or an oral hygienist at individual recall intervals. After the recession in the 1990s, resources for oral health promotion in the municipalities were limited. However, in a mid-sized Finnish city Laukaa, authorities wanted to still keep on prioritizing oral health promotion, and in 2008, the city launched a still on-going health education project the “Tooth Brushing School.” The “Tooth Brushing School” was based on ideology and methods used in Nexø. The aim of the project was to have all children less than 12 years of age and their parents/care givers living in Laukaa attend the “Tooth Brushing School.” In addition, in all Laukaa schools, oral health lessons were, and still are, organized every year with a specific theme. The themes have varied from healthy diet to dental caries. Examples of earlier themes are *Snacking; The Little Ones Follow the Big Ones' Example; *and *Good for Mouth, Good for You*. The oral health section of the municipal health services of Laukaa has also introduced its own hamster mascot, the *HAMSU *hamster (“HAMpaat ja SUu,” meaning “teeth and mouth”). The hamster appears in educational materials and on posters at the health center. There is also a website (http://www.hamsu.net/) where children can get more information about dental care together with their parents.

The aim of this study was to evaluate the outcome of a community-based oral health promotion project based on an individual as well as a public approach on schoolchildren's tooth brushing and other oral health behaviors. We hypothesized that schoolchildren's tooth brushing habits can be influenced by lessons at school and simple individual instructions, especially if the parents become involved.

## 2. Material and Methods

### 2.1. Oral Health Education

During the school year 2009-2010, an intense oral health promotion was carried out among all schoolchildren in Laukaa, Finland. The project was conducted by the oral health section of the Laukaa municipality. All children had dental education at school emphasizing mainly regular and careful tooth brushing. Some children in Laukaa, Finland had been invited to “Tooth Brushing School” in the summer of 2008 and even more of them during the school year 2009-2010. In Finland, all children under 18 years of age are entitled to dental care without charges by the municipality of their residence. The municipalities are required by the state to promote oral health. All expenses of this project were covered by the municipality of Laukaa.

In the “Tooth Brushing School,” children were first asked about their oral habits and then given a chance to “inspect” their own teeth using a hand mirror assisted by a dental assistant (PN). Children were demonstrated what a clean tooth looks and feels like and taught how to clean surfaces covered with plaque. If a child's oral habits were fine, they visited the “Tooth Brushing School” only once. In other cases, the child came to the “Tooth Brushing School” as often and as many times as needed, sometimes even every other week. Children were accompanied in the brushing school by their parents, who heard and saw what their children were taught.

### 2.2. Questionnaires

In the autumn of 2009, all children who were not absent from school answered a questionnaire on their oral health behavior. Children in grades 1 and 2 assisted by their parents answered the questionnaire at home and children in grades 3–6 at school. Teachers dealt out and collected the forms. A total of 1,185 out of 1,554 children (76%) answered the questionnaire (Table 1). In the analyses, the children were divided into two groups according to their grades: children in grades 1–4 and children in grades 5-6.

In the autumn of 2010, again all children who were not absent from school were asked to answer a questionnaire on their oral health behavior again. Out of 1,567 children, 1,293 (84%) answered this questionnaire. To compare the answers of the same groups of children in 2009 and 2010, for the analyses, the children were divided into two groups according to their grades: children in grades 2–5 and children in grade 6. The questionnaires in either year included no identification (IDs) of the children.

The questionnaires had been developed in the community and had not been validated. The questionnaires were similar to children in all grades. In 2009 and 2010, there were eight variables in the questionnaire with seven response alternatives describing the frequency of the behavior. The alternatives varied from “*three to four times a day or more frequently*” to “*less than twice a month or never*.” Questions in 2009 were “*How often do you brush your teeth; How often do you use dental floss; How often do you use tooth pick; How often do you use fluoride toothpaste; How often do you use nonfluoride toothpaste; How often do you use fluoride tablets; How often do you use xylitol products*;*”* and finally, “*How often do your parents help you with tooth brushing*?” In 2010, gender and school grade were also included in the questionnaire. A new question was “*Do your parents remind you of brushing your teeth*?” The alternatives given were “*yes*”, “*no*,” and “*I do not know*.” The following questions of 2009 were not included in 2010: “*How often do you use non-fluoride toothpaste*” and “*How often do your parents help you with tooth brushing*?” The question “How often do you use xylitol products?” was divided into two different questions as follows: “*How often do you use xylitol lozenges” and “How often do you use xylitol chewing gum*?”

### 2.3. Statistical Issues

The answers were recorded into two categories as follows. Brushing frequency was recoded into those brushing at least twice a day and the rest. Use of dental floss was recoded into those flossing at least 2-3 times a week and the rest. Use of fluoride toothpaste was recoded into those using it at least twice a day and the rest. Fluoride tablet use was recoded into those using them once a day and the rest. Use of any xylitol products, gum, or lozenge was recoded into those using them at least three to four times a day and the rest. Parental help was recoded into those children who were helped daily and the rest.

The answers to the questionnaire in 2009, and 2010 were compared using cross-tabulation. In 2009, the data had been collected combing the children in classes 1–4 and 5-6. In the year 2010, the class of each respondent was registered. To compare results in 2009 and 2010, the compared classes were 1–4 → 2–5 and 5-6 → 6. Those in class 7 attend a secondary school and did not participate in this study in 2010. The main results on oral health behaviors in 2009 and 2010 as well as in different grades in 2010 were presented graphically. Also, oral health behaviors of different groups were analyzed by cross-tabulation. Statistical significance of the differences between the groups was evaluated using Chi-squared test. Difference between the groups was considered statistically significant at *P* levels <0.05. Effect of different background factors was evaluated using binary logistic regression analysis, 95% confidence intervals. Goodness of fit of the model in these data was tested using Hosmer and Lemeshow test. SPSS (version 20.0, SPSS, Inc., Chicago, IL, United States) was used for the statistical analysis and for producing graphics.

### 2.4. Ethics

The data did not contain any personal identification of the patients; therefore, neither consent from the patients nor children, nor approval of an ethical committee was needed (Practices for Research Permits, Oulu University Hospital, Finland, 2009).

## 3. Results

**Figure 1:**
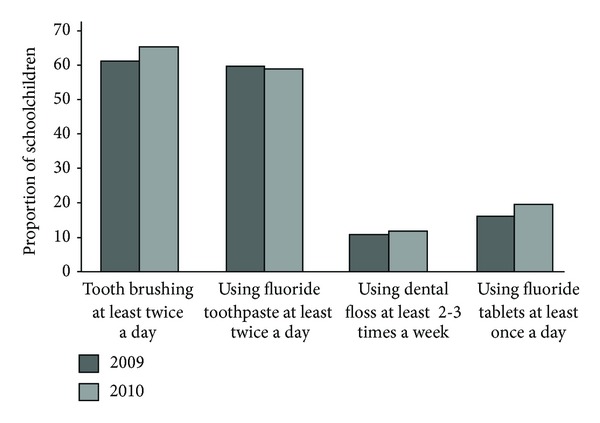
Self-reported oral hygiene habits before and after the intensive period of oral health promotion of schoolchildren.

**Figure 2:**
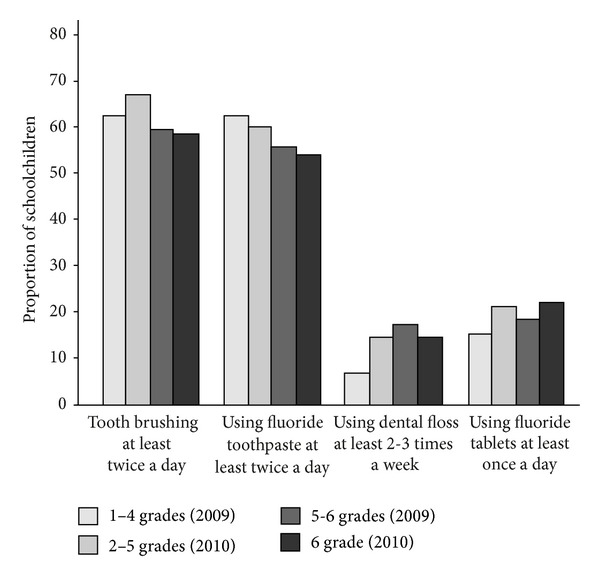
Oral hygiene habits of 1–4 and 5-6 grade pupils in 2009 and 2–5 and 6 grade pupils in 2010.

Tooth brushing and flossing frequency as well as use of fluoride tablets increased significantly among the schoolchildren during the study period 2009 and 2010 (*P* < 0.05). Frequency of using fluoride tooth paste practically remained the same (60.5% in 2009 versus 60.9% in 2010) (Figure 1). Both in 2009 and in 2010, younger children brushed their teeth and used fluoride toothpaste more frequently than the older ones (Figure 2). However, the older children (grades 5-6) used significantly more frequently dental floss and had fluoride tablets than the younger ones (grades 1–4) (*P* < 0.05) (Figure 2). The older children also used significantly more often xylitol products than the younger children did (*P* = 0.001). Children in lower grades got significantly more often daily parental help in tooth brushing in 2009 (21.4%), and parents reminded them of tooth brushing in 2010 (76.4%) significantly more often than older children (2.6% and 57.2%, (*P* < 0.001)).

The results in 2010 showed a slight trend of decreasing tooth brushing frequency by age (Table 2) both among girls and boys when investigated grade by grade. Nevertheless, use of dental floss, fluoride tablets, and xylitol lozenges increased until decreasing again between the grades 5 and 6. Significantly bigger proportion of children in the upper grades (55.3%) reported using xylitol chewing gum two times a day or more often compared to children in the lower grades (43.0%) (*P* < 0.001). Overall, girls brushed their teeth twice a day (72.0%) significantly more often than boys (57.3%). Girls also used toothpaste, dental floss, and xylitol gum significantly more often than boys (*P* < 0.05). Boys had significantly more often parental guiding than girls in every age group (*P* < 0.05).

According to the results in 2010, low tooth brushing frequency (daily or less frequently) was significantly affected by male gender and age (poorer towards grades 5-6). Tooth brushing education had a small impact on tooth brushing frequency, whereas parental reminding can be considered protective for children in lower grades (Table 3).

## 4. Discussion

Tooth brushing frequency increased significantly among the schoolchildren between the years 2009 and 2010, which shows that even a simple intervention targeted to groups and individuals of schoolchildren can be beneficial on their health behavior. Tooth brushing frequency increased more among younger children than among older children. We can also see a slight trend that tooth brushing frequency declines towards grades 5 and 6 among both girls and boys compared to younger children. Oral health habits of girls seem to be better than those of boys. These findings support the idea that oral health intervention should be given to all children but have focus on older children, especially boys. Education should be consistent, as suggested by Tolvanen et al. 2010 [7].

School health surveys are carried out in Finland every other year. In the 2008/2009 survey, 43% of 14–16-year-olds reported brushing their teeth twice a day or more often; the results show a slight improvement compared with the 2004/2005 survey (40%) [8]. Both in 2009 and in 2010, tooth brushing frequencies in Laukaa are well above those national averages, but again school-health surveys present mainly habits of older school-children. And it may well be that also in Laukaa the figures of this study group could be worse when the children grow older, if their oral health habits keep their worsening course even after grade 6. Anyhow, reason for delightful situation among the grades 2–6 in 2010 may be the beneficial effect of the years of oral health education organized in the Laukaa community on children's oral health. It has also been shown that municipalities which have stated goals and have a strong focus on caries prevention have improvement in caries experience data compared to communities without such strategies [9].

Over 1,000 children answered the questionnaire in 2009 and in 2010. The numbers are big and the response rates exceed 70% of the total age group in both years. High-response rate and big study group are strengths in this study. It is likely that some children were off school on the day the questionnaire was answered or sent home. Information about the number of children unable or refusing to answer the questionnaire was not available nor were the possible causes for refusals. This study is practice-based and was originally not designed to be a research. Therefore, the questionnaire used had not been validated and similar forms were used for children of different ages. This is a shortcoming in the present study. The children in grades 1 and 2 filled the forms at home with the help of their parents which makes the results more reliable for them. Even if parents may not be aware of their children's fear [10], they can be expected to know about 1st and 2nd graders tooth brushing habits. A deficiency of this study is also the lack of IDs of the respondents which hinders comparing results on the survey in 2009 and 2010 at individual basis, as well as having information on attendance in oral health promotion program at individual basis. Furthermore, it would have been valuable to compare DMFS or CPI values from patient records because the questionnaire does not tell anything about the quality of brushing, which is another main point besides frequency that “Tooth Brushing School” seeks to improve. Unfortunately, patient records contained no information of participation in “Tooth Brushing School” and again no IDs were collected, and thus oral health data could not be collected. Therefore, the effect on oral health could not be analyzed like it has been done in Nexø.

In our study, boys were lazier brushers than girls (57.2% and 72%). This supports the findings of the Finnish school health survey [8] and Kajaani, another city with a long history of continuous oral health promotion [11]. In Kajaani, 74% of 12-13-year-old girls and 66% of 15-16-year old girls reported brushing their teeth at least twice a day. For boys in those age groups, proportions of those brushing their teeth twice a day were 58% and 51%, respectively [11]. These brushing frequencies are well in accordance with our results. This again speaks for oral health promotion; low profile projects can be effective, when resources are limited for big ones.

It is interesting that the proportion of those using toothpaste twice a day is lower than the proportion of those brushing their teeth twice a day. It can be speculated why children do not always use toothpaste when brushing their teeth. According to Marinho et al. 2003 [12] and Axelsson et al. 1994 [13], it is beneficial for schoolchildren to brush their teeth twice a day with fluoride toothpaste. Here, it may be possible that not all children understood the question in the questionnaire about using fluoride toothpaste correctly. In the 2009 questionnaire, there were separate questions on the use of fluoride toothpaste and non-fluoride toothpaste, which could have confused children. In the 2010 questionnaire, the non-fluoride toothpaste alternative, however, was erased because most of the toothpastes in Finland contain fluoride. Therefore, the responses can be considered reliable.

Adair et al. 2004 [14] showed in their study that children whose parents had favorable attitudes towards controlling their children's tooth brushing had favorable oral health habits. In the present study, in 2009, a year after the “Tooth Brushing School” intervention started, children in grades 1–4 got significantly more often parental help than older children. Parental help may improve the level of oral hygiene and thus provide long-lasting benefits for the child. Unfortunately, the same question was not used in the 2010 questionnaire, and thus we cannot compare the responses in the two years. In 2010, 66.8% of the girls and 73.2% of the boys were reminded of brushing their teeth daily by their parents. In every age group, boys were more often reminded about tooth brushing by their parents than girls. According to Poutanen et al. 2007 [15], the parents' role model is extremely important to children and its effect is slightly stronger on boys than on girls. Children in grades 1–4 were significantly more often reminded about tooth brushing than children in grades 5-6. One explanation for this difference may be that children in grades 1-2 answered the questionnaire at home with their parents and children in grades 3–6 at school. The effect of parental reminding can be considered most effective for children in the low grades.

Xylitol products are commonly used in Finland. Over 30% of the children in Laukaa used xylitol products at least 3 to 4 times a day. Use of xylitol products was more common among older children and girls than younger children and boys. Regular use of both xylitol gum and candies is reported to reduce caries occurrence by about 50% compared with the control group [16]. Xylitol chewing gum has been shown to be equally effective with sealants in caries prevention [17]. Advantages of xylitol products are that they are freely available and can be bought without prescription. Use of xylitol is also cost-effective because it does not require oral health resources.

Analyzing an outcome by any oral health promotion project targeted to children is challenging; was the improvement or deterioration in oral health habits only coincidence, or caused by something else going on in the society, or simply by the growing age of a child? However, this should not hinder the health professionals from promoting oral health. To be effective, the promotion should not be only one project, but rather an on-going process like the one reported in Nexø. The confounding factors on oral health behavior were not analyzed here.

## 5. Conclusions

In conclusion, this study gives interesting information about oral health habits of 7–13-year-olds, when most of the previous studies in Finland and elsewhere have been conducted among older children. It can also be concluded that oral health promotion to schoolchildren is beneficial for their health behavior. Our findings emphasize a need of booster-programs targeted to 11-12-year-olds and especially boys. It is also noteworthy that intervention may be more effective on younger children (7–10-year-olds) compared to older children; maybe because of the activity of the parents, which should not be neglected as a resource in oral health promotion.

## Figures and Tables

**Table 1:** The total number of school children and number and proportion of the respondents in oral health surveys in 2009 and2010 in Laukaa, Finland.

Year	Number of respondents and total number schoolchildren *n*/total *n* (%)		
	Grades 1–4	Grades 5-6	Total
2009	702/1.029 (68.2)	483/525 (92.0)	1.185/1.554 (76.3)
2010	833/1.074 (77.6)	450/493 (91.3)	1.293/1.567 (82.5)

**Table 2:** Self-reported oral health behaviors of boys and girls in different grades of elementary school in Laukaa, Finland, 2010.

Grade	Tooth brushing ≥ 2 times/day		Use of fluoride tooth paste ≥ 2 times/day		Parents reminding of tooth brushing daily		Use of dental floss ≥ 2-3 times/week		Use of fluoride tablets daily		Use of xylitol chewing gum ≥ 3-4 times/day		Use of xylitol lozenge ≥ 3-4 times/day	
	Girls	Boys	Girls	Boys	Girls	Boys	Girls	Boys	Girls	Boys	Girls	Boys	Girls	Boys
1	71.8	60.0	69.2	58.2	88.9	91.8	0.0	0.0	9.6	13.5	17.2	15.5	10.4	10.1
2	67.0	68.0	64.8	66.0	90.5	92.7	1.0	3.1	10.6	16.7	20.8	11.5	16.0	6.2
3	76.4	62.8	68.9	53.8	56.2	60.6	17.3	15.4	21.0	25.6	34.3	29.3	16.0	17.6
4	78.7	48.5	67.8	41.1	62.9	64.3	27.0	10.5	30.2	22.5	34.1	29.2	11.5	20.9
5	75.4	52.5	62.9	49.0	59.3	64.4	24.8	14.0	20.7	19.1	43.2	31.7	9.4	18.0
6	63.6	52.7	61.5	47.2	41.8	64.2	20.4	7.7	17.9	25.5	33.0	21.8	11.0	11.3

Total	72.0	57.3	65.8	52.6	66.8	73.2	14.8	8.2	17.9	20.4	30.4	23.0	12.3	13.8
*P*	**<0.001**		**<0.001**		**0.040**		**<0.001**		0.244		**0.003**		0.263	

**Table 3:** Binary logistic regression analysis on the ODDs by gender, grade at school, received tooth brushing education, and parental reminding on low tooth brushing frequency (daily or less frequently).

Variable	OR	95% CI		*P*
		Lower	Upper	
Male gender	1.94	1.491	2.512	<**0**.**001**
Classes 5-6	1.39	1.049	1.833	**0.022**
Tooth brushing education	0.83	0.635	1.083	0.170
Parental reminding	0.69	0.503	0.956	**0.026**

Hosmer and Lemeshow *κ* ^2^ = 3,554, df = 7, *P* = 0.829.
